# Self-transport and self-alignment of microchips using microscopic rain

**DOI:** 10.1038/srep14966

**Published:** 2015-10-09

**Authors:** Bo Chang, Ali Shah, Quan Zhou, Robin H. A. Ras, Klas Hjort

**Affiliations:** 1 Department of Applied Physics, Aalto University, FI-00076, Espoo, Finland; 2 Department of Engineering Sciences, Uppsala University, SE-75121, Sweden; 3 Department of Micro- and Nanosciences, Aalto University, FI-00076, Espoo, Finland; 4 Department of Electrical Engineering and Automation, Aalto University, FI-00076, Espoo, Finland

## Abstract

Alignment of microchips with receptors is an important process step in the construction of integrated micro- and nanosystems for emerging technologies, and facilitating alignment by spontaneous self-assembly processes is highly desired. Previously, capillary self-alignment of microchips driven by surface tension effects on patterned surfaces has been reported, where it was essential for microchips to have sufficient overlap with receptor sites. Here we demonstrate for the first time capillary self-transport and self-alignment of microchips, where microchips are initially placed outside the corresponding receptor sites and can be self-transported by capillary force to the receptor sites followed by self-alignment. The surface consists of hydrophilic silicon receptor sites surrounded by superhydrophobic black silicon. Rain-induced microscopic droplets are used to form the meniscus for the self-transport and self-alignment. The boundary conditions for the self-transport have been explored by modeling and confirmed experimentally. The maximum permitted gap between a microchip and a receptor site is determined by the volume of the liquid and by the wetting contrast between receptor site and substrate. Microscopic rain applied on hydrophilic-superhydrophobic patterned surfaces greatly improves the capability, reliability and error-tolerance of the process, avoiding the need for accurate initial placement of microchips, and thereby greatly simplifying the alignment process.

Capillary self-alignment is a crucial step of many emerging technologies in micro- and nanosystem integration, such as fluidic self-assembly^1^,^2^, hybrid microassembly^3^,^4^, and solder self-assembly^5^,^6^. Minimizing surface energy is the fundamental principle behind capillary self-alignment, where the gradient of the surface energy is designed to drive the microchips towards desired locations. Capillary self-alignment has been demonstrated previously, including massively parallel microassembly^7^,^8^,^9^, integration of relatively complicated 3D structures^10^,^11^,^12^, fluidic self-assembly of GaAs blocks^13^ and optoelectronic devices^14^. Impressive results have been reported, e.g. 62,500 chips assembled in 45 seconds with sub-micrometer precision^2^ and implementation of automated reel-to-reel fluidic self-assembly machine^15^. Recently, a so called hybrid microassembly technique combining robotic pick-and-place with capillary self-alignment was reported^3^, which achieved more deterministic results and new designs that are not possible with self-assembly alone. An industrial pilot demonstration achieved high yield (98%) and micrometer-precision at a speed of over 40,000 units per hour^16^.

Although capillary self-alignment has attractive features, it requires placement of the microchips on the receptor site, either accurately, by e.g. robotic pick-and-place^3^ or randomly, by e.g. fluidic agitation^9^. The capillary self-alignment takes place only when the microchips come in contact with the liquid self-alignment medium on the receptor sites, and significant overlap between the microchips and the receptor sites is always required, usually at least 50%, to achieve stable self-alignment at good yield^3^,^17^. Despite many impressive results, capillary self-alignment has never been demonstrated with microchips initially placed outside the receptor sites. Moreover, the capillary self-alignment process is very sensitive to the volume of the liquid delivered to the receptor sites. Too much liquid could lead to overflow from the receptor site onto the substrate, which is one of the main causes for failure in capillary self-alignment. On the other hand, too little liquid causes inefficient wetting and dry contacts, which increases the friction force between the microchips and the receptor sites and leads to misalignment.

In this paper, we report a method to achieve capillary self-transport and self-alignment of microchips that requires no initial overlap between the microchips and the receptor sites and importantly is much more robust to the liquid volume. The *self-transport* moves the chip initially placed outside of the receptor site in contact with the receptor site, and the *self-alignment* aligns the chip according to the geometry of the receptor site. For simplicity, we refer to the whole process as just *self-transport* in this paper if not otherwise specified. We achieve the self-transport by using patterned superhydrophobic-hydrophilic surfaces and microscopic rain. The substrate is superhydrophobic black silicon coated with a fluoropolymer and has hydrophilic silicon patterns. Microscopic rain is used as a medium for capillary self-transport, being a cloud of micron-sized droplets suspended in the air. When the rain falls onto a surface, hundreds of microscopic droplets are deposited on the surface. The process is similar to rain, but at a much smaller scale. Therefore, in this paper, this particular process is referred to as ‘microscopic rain’. To understand the experimental results, we simulate the capillary self-transport process with Surface Evolver^18^, which uses the gradient descent method to evolve the liquid surface toward its minimal energy.

A schematic of the procedure of the capillary self-transport can be seen in Fig. 1(a–f). A microchip is coarsely placed outside a matching sized receptor site. Then water droplets are delivered to the substrate in the form of microscopic rain where the droplets fall on the whole substrate due to gravity or air flow. The deposited water droplets form a continuous film on the hydrophilic receptor site due to its low contact angle, and form discrete droplets on the superhydrophobic substrate. When sufficient amount of water droplets are deposited, the droplets between the chip and the receptor site grow to a size that will establish a meniscus between the chip and the receptor. This meniscus and the water film on the receptor coalesce which then reduces its total surface energy by pulling the chip towards the receptor and finally align with the receptor site. The total surface energy of the meniscus as a function of the gap is illustrated in the schematic plot (Fig. 1(g)). Gap is defined as the distance between the chip (red) and the pad (green). The surface energy of the meniscus decreases as the chip moves from its initial position (Fig. 1(c)) towards the receptor site. The surface energy is minimized and the meniscus reaches its equilibrium state when the chip is perfectly aligned with the receptor site (Fig. 1(f)). The self-transport happens extremely fast (Supplementary Movie S1) once the droplets on the edge of the chip are merged with the water film on the receptor site, and it takes only a few to tens of milliseconds for the chip to move from its initial position (Fig. 1(c)) to its target position (Fig. 1(f)). Due to the superhydrophobicity of the substrate, once a droplet is in the hydrophilic receptor site, the droplet will be confined inside that area.

## Results

### Numerical simulations

**Figure 2:**
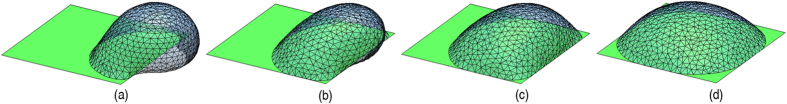
Simulation of a water droplet (1 nL) moving on a 200 μm × 200 μm hydrophilic/superhydrophobic (50°/170°) patterned surface: (**a**) a water droplet is placed on a patterned surface, where half of the droplet rests on the hydrophilic receptor site and other half is on the less wettable substrate; (**b,c**) the droplet moves towards more hydrophilic area; (**d**) the droplet reaches its equilibrium state.

Capillary self-transport process is based on the principle of minimizing surface energy of liquid droplets. The self-transport process has been numerically investigated using Surface Evolver. Figure 2 shows a simulation of a water droplet moving on a hydrophilic/superhydrophobic patterned surface, where part of the droplet is on the hydrophilic receptor site and other part is on the superhydrophobic substrate. Due to the surface energy gradient, the droplet moves towards the more hydrophilic area until it reaches its equilibrium state.

To understand the self-transport process, three elements were taken into consideration in the simulation: a hydrophilic receptor site with hydrophobic substrate, a droplet of water and a chip placed at a specific distance from the receptor site. The dimension of the chip and the receptor site are both 200 μm (L) × 200 μm (W), and both the gravitational force and friction are considered negligible. The gravitational force of the chip can be neglected because the size of the chip (200 μm) is much smaller than the capillary length (2.7 mm) for water at standard temperature and pressure^19^. The friction force between the chip and the substrate can be calculated using the equation *F*_*f*_ = *μ* * *F*_*N*_, where *μ* is the coefficient of friction and *F*_*N*_is the normal force. Bhushan *et al.*^20^ measured the coefficient of friction on surfaces with different wetting properties using atomic force microscopy, and showed that friction force on a hydrophobic surface is much smaller than the friction force measured on a hydrophilic surface, e.g. the coefficient of friction on a lotus leaf (contact angle with water: 150°) is 0.05, whereas the coefficient of friction on a hydrophilic magnolia leaf (contact angle with water: 80°) is 0.1. In our simulation, *F*_*N*_ is simply the weight of the chip, which is around 0.014 μN. Assuming that the coefficient of the friction of a hydrophobic surface is approximately 0.05^20^, the estimated friction force should be less than 1 nN, which is about 1,000 times smaller compared to the restoring force (several micro-newton) acting on a droplet with volume of 0.9 nL reported in our previous work^21^. Therefore, the friction force can be safely neglected in the simulation.

**Figure 3:**
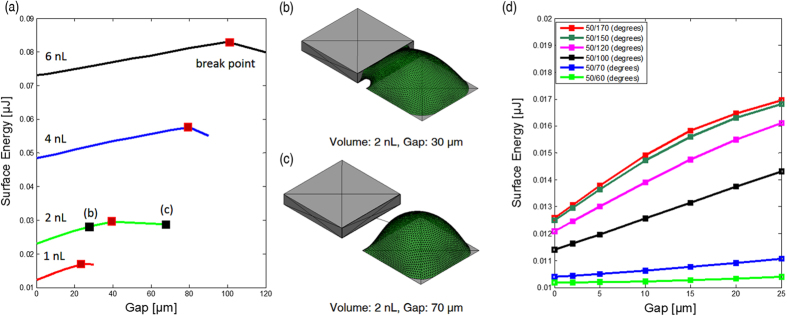
(**a**) Surface energy as function of gap size with respect to the volume (red marks represent break points where the meniscus starts to break up and black mark on the green curve represents case (**b**,**c**), respectively); (**b**,**c**) simulation of self-transport with a chip placed 30 μm and 70 μm away from a receptor site; (**d**) surface energy as function of gap size with respect to the wetting contrast.

In order to find boundary conditions for self-transport and investigate how far a chip can be placed outside a receptor site, we simulated the process by adjusting the volume of the droplet. The contact angles of the receptor site and the substrate are 50° and 170°, respectively. Figure 3(a) shows the relationship between the gap and total surface energy with respect to the volume of liquid. The break point in the energy curve (highlighted with a red mark) indicates that the water meniscus starts to break up when the gap reaches its limit. The volume has clear influence on the maximum gap between the chip and receptor site. The larger the volume of the liquid is, the greater the allowed gap. With an increase of volume to 6 nL, the chip can be misplaced up to 100 μm away the receptor site. On the other hand, as the volume decreases to 1 nL, the maximum gap between the chip and the receptor site is reduced though still a respectable 25 μm. Figures 3(b,c) represent respectively successful and failed self-transport with the same volume of liquid, the upper one shows a chip placed within the gap limit and there is enough liquid to connect the chip and the receptor site; the other case represents a chip placed beyond the gap limit and a water bridge cannot be established.

To understand the influence of the wetting contrast on capillary self-transport process, 6 sets of wetting contrast were simulated. The volume of the droplet was kept as 1 nL for all the simulations. The contact angle of water on the receptor site was fixed at 50°, and the contact angle on the substrate varied from 60° to 170°. Figure 3(d) shows the relation between the surface energy and the gap with respect to the wetting contrast. The surface energy decreases as the gap narrows. Therefore, in theory, when a droplet is placed on a patterned surface, where part of the droplet is sitting on the hydrophilic receptor site and part of it is on the less wettable substrate, even with very small wetting contrast, e.g. 50° on receptor site and 60° on substrate, the droplet will move towards more hydrophilic area, provided that the gap is within the maximum allowable range for the particular configuration. However, when the substrate is hydrophilic and the wetting contrast is small, the self-transport most likely cannot be achieved due to (1) the increasing friction force between the chip and hydrophilic surface compared to the friction force between a chip and a superhydrophobic surface; (2) the lower the wetting contrast is, the more flat the energy curve becomes, and the flat energy curve leads to low restoring force. A case of failed self-alignment is demonstrated using hydrophilic/hydrophobic patterned surface (Supplementary Figure S1), which consists of hydrophilic silicon receptor site (water contact angle: 50°) and hydrophobic fluoropolymer coated silicon substrate (water contact angle: 100°). Despite the large wetting contrast between the receptor site and the substrate and the hydrophobicity of the substrate, the self-alignment failed since the friction force between the chip and the substrate becomes much larger and stops chip from moving. Therefore, the key factors to achieve reliable capillary self-transport are large wetting contrast between the receptor site and the substrate, and the superhydrophobicity of the substrate.

### Experimental demonstrations

**Figure 4:**
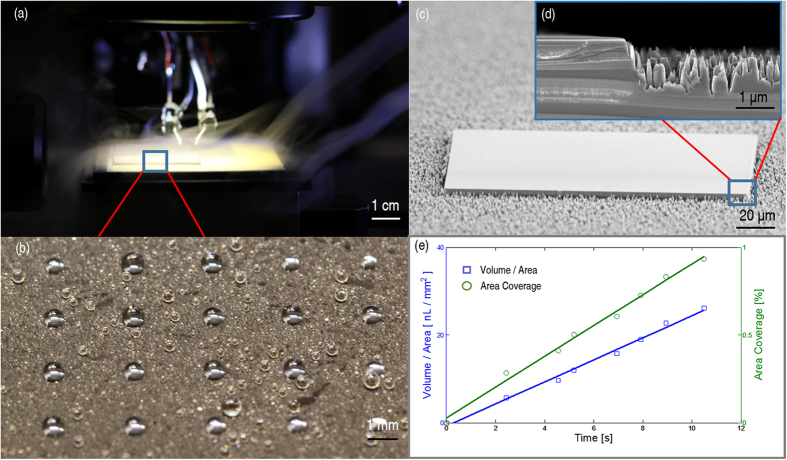
Experimental set-up and test sample: (**a**) Robotic micro-handling tool with microscopic rain; (**b**) microdroplets deposited on a hydrophilic/superhydrophobic patterned surface; (**c**) scanning electron micrograph of a silicon receptor site on black silicon coated with fluorocarbon polymer; (**d**) cross-sectional view of a patterned surface (planar silicon receptor site on left and spiky fluoropolymer coated black silicon on right); (**e**) volume per unit area and coverage area as function of microdroplet deposition time.

Experimental tests have been carried out to investigate the proposed self-transport process with microscopic rain (Fig. 4(a)). The test sample consists of silicon receptor sites and black silicon substrate coated with fluorocarbon polymer. Contact angle hysteresis is an important factor for self-transport. As the water droplet on the hydrophilic receptor site reaches the chip that was placed on the superhydrophobic substrate, the water contact angle on one side of the droplet is the receding contact angle on the superhydrophobic substrate, and the contact angle on the other side of the droplet is the advancing contact angle on the receptor site. To achieve self-transport, the receding contact angle on the substrate needs to be significantly larger than the advancing contact angle on the receptor site. In our experiments, the measured advancing/receding contact angle on the substrate and the silicon receptor site are 174°/168° and 52°/28°, respectively (Supplementary Figure S2). The receding contact angle on the substrate is much greater than the advancing contact angle on the receptor site, which is the key for successful self-transport. Due to the large wetting contrast and the superhydrophobicity of the substrate, the water droplets can be confined inside the hydrophilic silicon receptor sites as shown in the Fig. 4(b). Scanning electronic micrographs of a silicon receptor site on black silicon substrate coated with fluorocarbon polymer are shown in Fig. 4(c,d). The combination of the black silicon nanostructures and the low surface energy fluoropolymer contributes to the superhydrophobicity of the substrate. Microscopic rain was produced using an ultrasonic humidifier, which contains a reservoir of water and an oscillating plate. The deposition process was recorded with a high-speed video camera and the droplets were detected and volume of the droplets was calculated with a machine-vision algorithm. The algorithm was implemented in MATLAB. The diameter of the droplets is in the range of 1–5 μm. Due to the superhydrophobicity of the surface, the droplets can be assumed as spheres and the volume can be calculated based on the diameter observed from the video camera. Figure 4(e) shows the volume of the microdroplets per unit area and the percentage of the area covered by microdroplets as function of the deposition time. The results indicate that both the volume per unit area and the coverage area increase linearly with deposition time.

**Figure 5:**
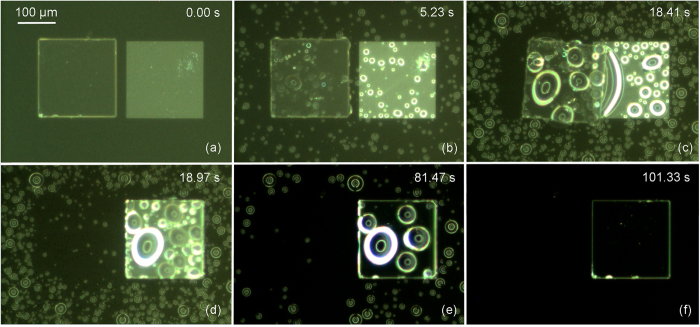
Self-transport of a 200 μm × 200 μm × 30 μm SU-8 chip on a matching sized silicon receptor site with superhydrophobic substrate using microscopic rain: (**a**) a chip is placed next to a receptor site with a gap of 25 μm; (**b**) microscopic rain is delivered to the assembly site; (**c**) a liquid meniscus forms between the receptor site and the chip; (**d**) the chip aligns with the receptor site due to the capillary force; (**e**) droplets are evaporating; (**f**) water has evaporated leaving the surface dry. The images are snapshots from Supplementary Movie S2.

In the tests, a 200 μm × 200 μm × 30 μm SU-8 chip is coarsely placed on the superhydrophobic substrate next to a matching sized hydrophilic receptor site using a robotic micro-handling tool as shown in Fig. 4(a). Microscopic rain was introduced to the assembly site to create the microscopic droplets for the capillary self-transport. Examples of the tests are shown in Fig. 5(a–f). The gap between the chip and the receptor site is about 25 μm. The microscopic droplets were accumulating on both the top of the chip, the receptor site and the substrate. The microscopic rain was delivered for about 18 seconds and the amount of the droplets forming a water bridge between the chip and the receptor site was estimated as 1.5 nL. Once the water bridge was formed, the self-transport was extremely fast and took only about 0.5 seconds for the chip to move from its initial position to where it is perfectly aligned with the receptor site (Fig. 5(c,d)). After the self-alignment occurred, it took tens of seconds for the droplets to be evaporated, leaving the surface dry (Fig. 5(d–f)). Capillary self-transport is also possible when the chip was placed next to a receptor site with both linear positioning error and rotational positioning error (Supplementary Figure S3). The capillary self-transport tests have been repeated for 15 times with gap sizes ranging from 10 μm up to 90 μm. Among the tests, 14/15 tests resulted in successful self-alignment. One exception was due to insufficient droplet deposition, leading to too fast droplet evaporation (Supplementary Figure S4). This result is in sharp contrast to cases where the substrate is not superhydrophobic (Supplementary Figure S1), and self-alignment of a chip placed outside its receptor site is not possible. We found two critical parameters to allow successful self-transport. Firstly, it is important to supply sufficient liquid during the self-transport process. In case of insufficient liquid deposition during the final stage of the self-transport, the chip aligned only partially (Supplementary Figure S4). The second critical parameter is the gap size. When the minimum gap was 90 μm, the large droplets on the receptor site and the chip led to a flip-chip mechanism in the self-transport (Supplementary Figure S5). The droplets contacted each other and lifted the chip up-side-down before drying and aligning it on the receptor site. This sets a maximal limit for a stable self-transport that drags the chip towards the receptor site.

## Discussion

Successful capillary self-transport is based on two key factors, namely the superhydrophobicity of the substrate and the continuous supply of microscopic rain. Superhydrophobicity is important for a couple of reasons. Firstly, the strong dewetting behavior of the superhydrophobic surface prevents droplet spreading even when the droplet is in contact with the substrate. This greatly enhances the stability of capillary self-alignment process, because the process becomes less sensitive to the amount of liquid delivered onto the assembly site. In this paper, the volume used for self-transport spans from 1 nL to 10 nL, which is a larger range compared to the earlier results of capillary self-alignment^3^, where the volume of liquid had to be controlled very carefully in the range of 0.97 nL–3 nL to allow for successful self-alignment. Secondly, the dewetting capability of the substrate is also critical to achieve the self-transport, compared to earlier results^7^ where self-alignment can only reliably occur when the chip is placed on top of the receptor site and the initial misplacement is less than half of the size of the chip. Thirdly, less friction force and adhesion force are generated when a superhydrophobic surface is in contact with other surfaces, because superhydrophobic surface consists of micro- and nano-structures and reduces the contact area. Therefore, with the microchip placed outside the receptor site, the capillary force can easily overcome the friction force and adhesion force between the chip and the substrate to achieve the alignment. The second key for capillary self-transport is the continuously deposition of tiny water droplets. Previously it was challenging to reliably deposit water on superhydrophobic surface due to the very large contact angle of the surface. The droplets will bounce away on superhydrophobic substrate^22^ since the momentum of the droplet is significant compared to the adhesion force between the droplet and the substrate. Here we provide a robust strategy to reliably deposit liquid onto a superhydrophobic surface using a large number of micro-sized droplets or microscopic rain, where the capillary force scales favorably compared to the momentum in smaller scales.

Our experimental results follow the simulations of the maximum gap for the most common cases where capillary force drags the chip towards the receptor site. Surprisingly, we noticed that self-transport is even possible when the gap is greater than estimated maximum allowable gap from the simulation. This is due to a more complicated self-transport scenario involving the accumulation of droplets on both the top sides of the chip and the receptor site, which touch each other and leads to flipping of the chip and followed by the ultimate self-alignment (Supplementary Figure S5).

Additionally, despite the whole process from the deposition of the microscopic droplets to the completion of the evaporation takes tens to hundreds of seconds, it will not be a limiting factor in real applications where the self-transport can be done in a parallel manner and the deposition and evaporation can take place while the substrate was under transportation before or after parallel self-transport occurs, e.g. in roll-to-roll or sheet-to-sheet processes. Furthermore, capillary self-transport would work with different sizes of the chips. According to the scaling law, capillary force plays a more dominant role than gravity when the size of the chip is smaller than the capillary length (2.7 mm), therefore chips less than 2 mm are preferred for capillary self-transport. The process reported in this paper could also be applicable to multiple chips alignment, where the distances between chips should be reasonable so that only one chip is sufficiently close to a particular receptor site to allow deterministic self-transport.

In conclusion, this paper demonstrates that microscopic rain and hydrophilic/superhydrophobic patterned surfaces cannot only be used for capillary self-alignment, but they also can tolerate extremely large placement errors beyond the current state of the art through the self-transport process. Additionally, hydrophilic/superhydrophobic patterned surfaces also greatly enhance the reliability and robustness of the self-alignment process.

## Methods

### Fabrication of black silicon patterned surface

Single-side-polished silicon wafer was used as a substrate for the fabrication of patterned hydrophilic/superhydrophobic surfaces. Firstly, 700 nm silicon dioxide hard mask material was deposited at 61 nm/min using plasma enhanced chemical vapor deposition (Oxford Instruments Plasmalab80Plus). Standard photolithography with positive tone resist was used to spin and pattern the photoresist (AZ5214E) on top of silicon dioxide. Silicon dioxide etching was performed in buffered hydrofluoric acid (BHF) heated at 30 °C for 2 min, where the photoresist served as a mask material for receptor sites. Photoresist removal was done in an acetone bath with ultrasound for 5 min. In the next step, black silicon was formed in Oxford Instruments Plasmalab system100 cryogenic inductively coupled plasma reactive ion etching (ICP-RIE) for 7 min using optimized parameters (40 sccm of SF_6_, 18 sccm of O_2_, forward power of 6 W, ICP power of 1000 W, temperature of −110 °C, pressure of 10 mTorr and helium backside cooling)^23^. A thin layer (around 50 nm) of low surface energy fluoropolymer coating was deposited using CHF_3_ in a reactive ion etcher (Oxford Instruments Plasmalab80Plus). In the final step, the fluoropolymer coating from receptor sites was lifted-off in BHF for 2 min.

### Simulation method

Capillary self-transport is based on the principle of minimum surface energy of liquid droplet, where the gradient of potential drives the parts toward desired alignment locations. A droplet is in stable equilibrium when its surface energy is at a minimum. When biases are introduced, the restoring force that acts on the chip and directs the meniscus to the desired position can be calculated by:


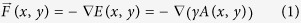


where *E* is the surface energy of the liquid meniscus, γ is the surface tension and *A* is the area of the surface between liquid and air, *x* and *y* are the biases along corresponding axes. We use Surface Evolver^18^ to find the static equilibrium for liquid medium by evolving the surface using the gradient descent method. Surface Evolver breaks the surface of the liquid droplet into smaller elements, and minimizes the surface energy of each element, by optimizing the location of each vertex.

### Experimental setup

To study the capillary self-transport process, the tests were carried out using a hybrid microassembly platform. The platform consists of a customer designed microgripper, four motorized stages (2 PI/M-111.1DG, 1 PI/M-122.2DD, and 1 PI/M-404.8PD), an ultrasonic humidifier (Bionaire Ultrasonic Compact BU1300W-I) and two microscopes (Edmund/VZM1000i) connected to two video cameras (Imperx/IGV-B1620 and IGV-B1320). The microgripper is used to place the microchip next to the receptor site. The motorized stages are responsible for moving the microgripper (z-axis) and the sample carrier (x, y and z axes). The rain-induced microdroplets are produced by the ultrasonic humidifier, which contains a reservoir of water and an oscillating plate. The oscillating plate is attached to the bottom of the water reservoir, and it vibrates and generates intense ultrasonic vibration. The vibration is then transported to the boundary layer between water and air. The high frequency compression and decompression of the water above the oscillating plate causes cavitation near the water surface. Crossed capillary waves are then formed, where very small droplets dispersed into air from the wave. To observe the test procedure and results, two microscopes (Edmund/VZM1000i) are installed, one on the top and one to the side. A high-speed camera (Phantom Miro M310) was used to record the deposition of microscopic droplets and the fast moment of the self-transport process (Supplementary Movie S1).

## Additional Information

**How to cite this article**: Chang, B. *et al.* Self-transport and self-alignment of microchips using microscopic rain. *Sci. Rep.* **5**, 14966; doi: 10.1038/srep14966 (2015).

## Supplementary Material

Supplementary Figures

Supplementary Movie S1

Supplementary Movie S2

## Figures and Tables

**Figure 1:**
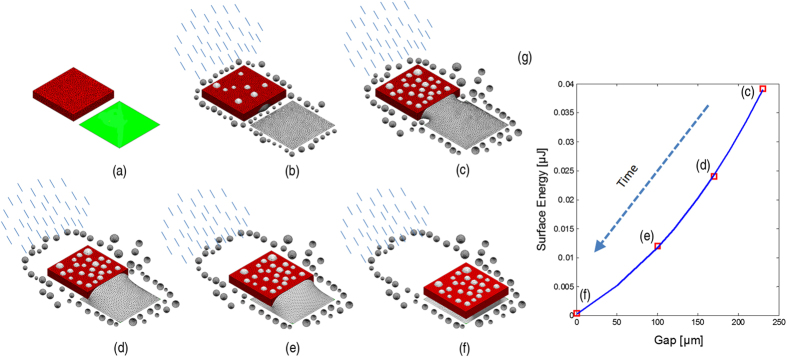
The procedure of capillary self-transport using microscopic rain and the schematic plot of the surface energy as function of the gap between chip and receptor site: (**a**) a chip (red) is placed outside a receptor site (green) with the same shape and size; (**b**) then microscopic rain is delivered onto the assembly site and water droplets are accumulating on the hydrophilic receptor site and superhydrophobic substrate; (**c**) next, water droplets are in contact with the edge of the chip and a water meniscus is formed between the chip and the receptor site; (**d–f)** the chip is dragged towards the receptor site and finally aligned with the receptor site; (**g**) the decrease in surface energy of meniscus drives the microchip to move from the position in (**c–e**) to finally reach the minimum at (**f**).
