# Supplementary material for: Self-transport and self-alignment of microchips using microscopic rain
Source: Sci Rep. 2015 Oct 9;5:14966. doi: 10.1038/srep14966 (PMC4598810; doi:10.1038/srep14966)
Supplement: Supplementary Figures [file srep14966-s1.doc]

**Supplementary Information**

**Self-transport and self-alignment of microchips using microscopic rain**

Bo Chang^1, 2, *^, Ali Shah^3^, Quan Zhou^4, *^, Robin H. A. Ras^1^, Klas Hjort^2^

^1^Department of Applied Physics, Aalto University, FI-00076, Espoo, Finland, ^2^Department of Engineering Sciences, Uppsala University, SE-75121, Sweden, ^3^Department of Micro- and Nanosciences, Aalto University, FI-00076, Espoo, Finland, ^4^Department of Electrical Engineering and Automation, Aalto University, FI-00076, Espoo, Finland.

^*^ Corresponding authors: Bo Chang (email: [bo.chang@aalto.fi](mailto:bo.chang@aalto.fi)) and Quan Zhou (email: quan.zhou@aalto.fi)

**Supplementary Figures**

**
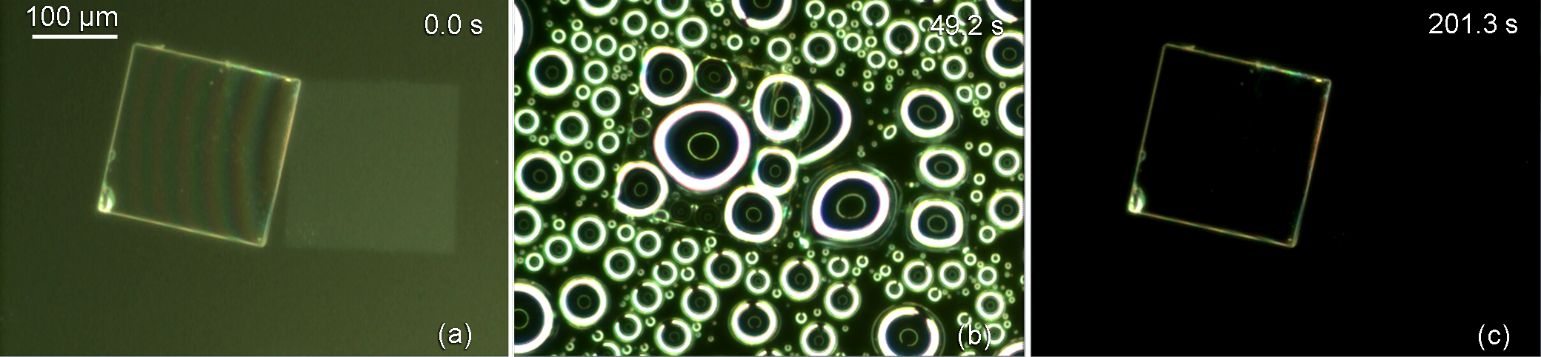
**

**Supplementary Figure S1.** Failed self-alignment of a 200 µm × 200 µm × 30 µm SU-8 chip on a matching sized silicon receptor site with hydrophobic fluoropolymer coated substrate using microscopic rain due to the large adhesion on the moderately hydrophobic surface: (a) a chip is placed on a hydrophilic/hydrophobic (50°/100°) patterned silicon surface; b) rain-induced microdroplets are delivered to the assembly site and water droplets are accumulated on the surface of the hydrophilic pad and hydrophobic substrate; c) the chip stays at its initial position without any movement towards receptor site.


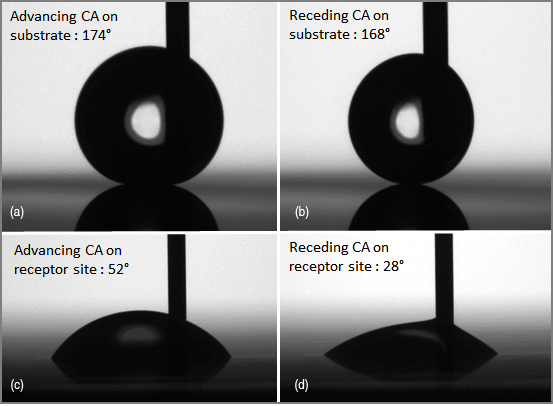


**Supplementary Figure S2.** Advancing and receding contact angle of water droplet on superhydrophobic black silicon substrate and silicon receptor site: (a) advancing contact angle of water droplet on black silicon substrate (174°); (b) receding contact angle of water droplet on black silicon substrate (168°); (c) advancing contact angle of water droplet on silicon receptor site (52°); (d) receding contact angle of water droplet on silicon receptor site (28°).


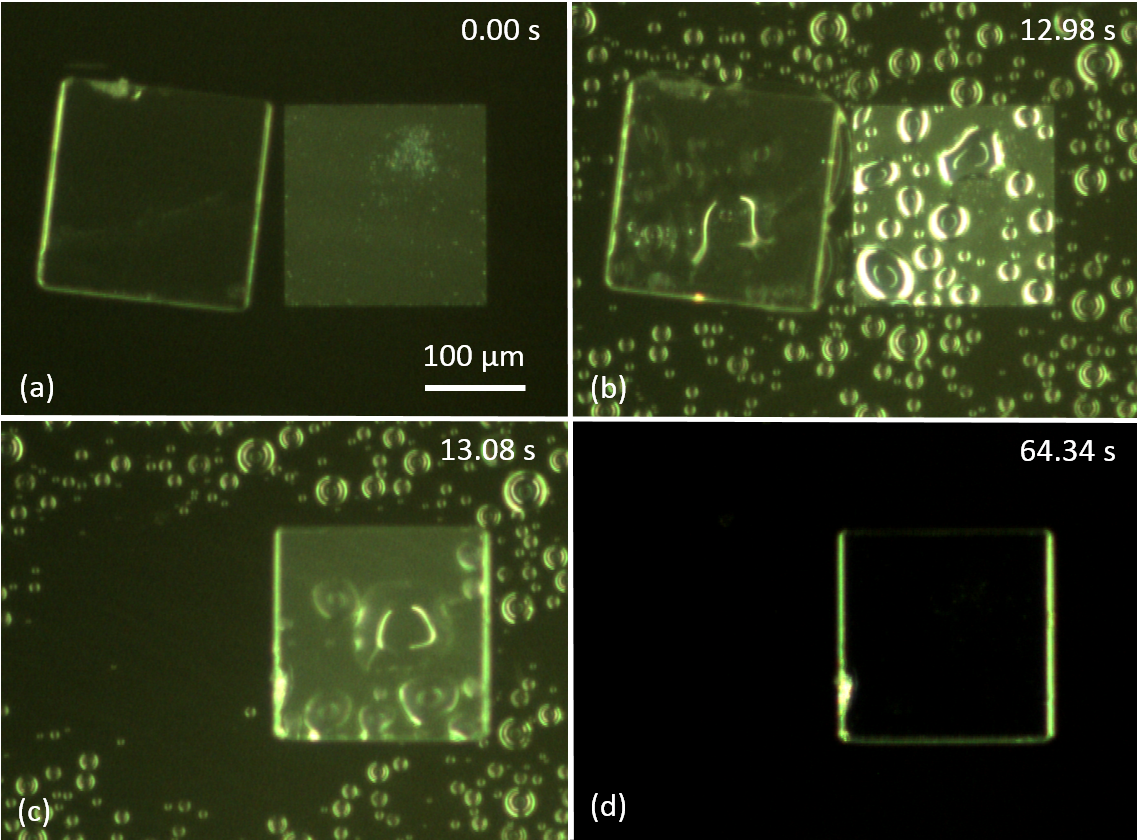


**Supplementary Figure S3.** Successful capillary self-transport of a 200 µm × 200 µm × 30 µm chip placed next to a receptor site with both linear positioning error and rotational positioning error: (a) a chip is placed next to a receptor site with minimum gap of 15 µm and rotational error of 8°; (b)-(c) microscopic rain is delivered to the assembly site and the chip aligns to the receptor site due to the capillary force; (d) droplets have evaporated leaving the surface dry.


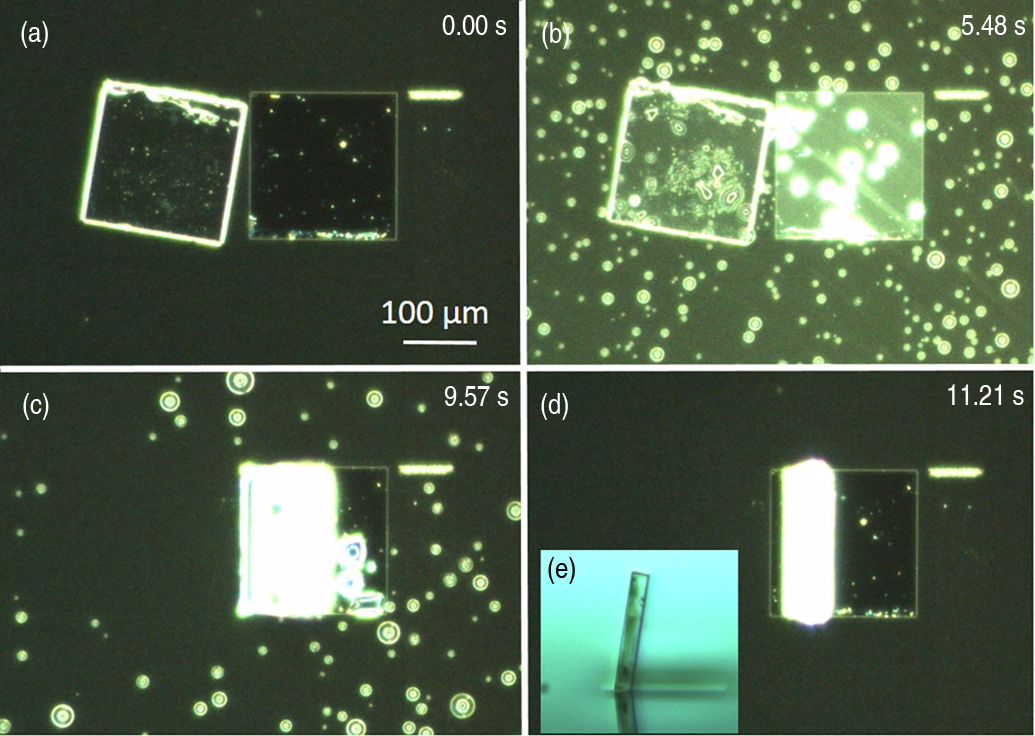


**Supplementary Figure S4.** Partial self-alignment due to insufficient droplets deposition and fast droplet evaporation: (a) a 200 µm × 200 µm × 30 µm chip is placed next to a receptor site; (b) microscopic rain is delivered to the assembly site; (c) the chip moves towards the receptor site but it is tilted; (d)-(e) droplets have evaporated before final self-alignment is achieved (observed from top view (d) and side view (e)).


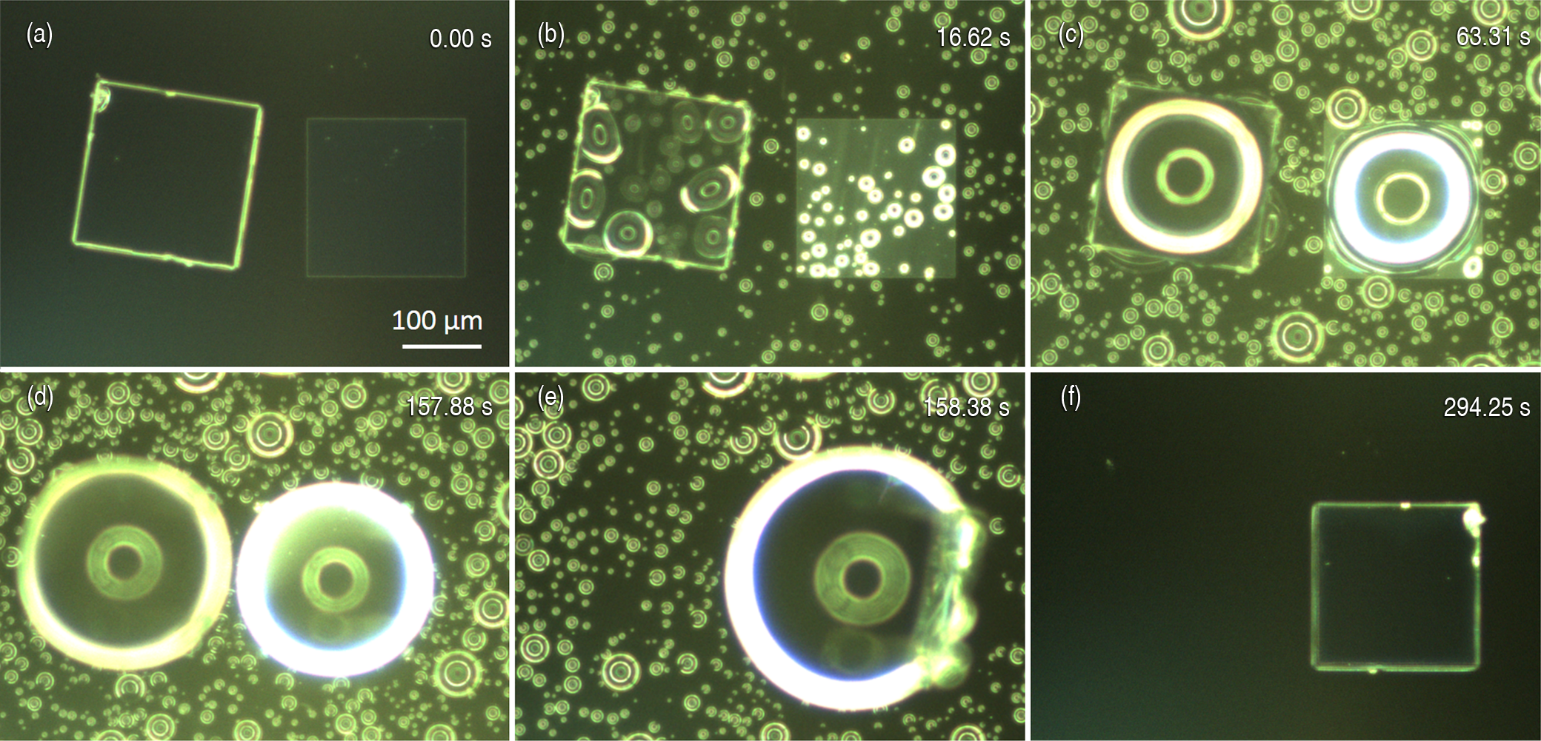


**Supplementary Figure S5.** Successful capillary self-transport of a 200 µm × 200 µm × 30 µm SU-8 chip placed next to a receptor site with an extreme large placement error: (a) a chip is placed next to a receptor site with minimum gap of 90 µm; (b)-(c) microscopic rain is delivered to the assembly site and water droplets are accumulating on both the top of the chip, the receptor site and the substrate; (d)-(e) The droplet is growing larger and larger on the top of the chip and on the receptor site and eventually two droplets are merged into one droplet and the chip is lifted up-side-down by the droplet and aligned to one edge of the receptor site; (f) water has evaporated and the chip is flipped and aligned with the receptor site.

**Supplementary Movies**

**Supplementary Movie S1.** Slow motion of self-transport and self-alignment.

**Supplementary Movie S2.** Self-transport and self-alignment of microchips using microscopic rain: (a) a chip next to a receptor site; (b) microscopic rain; (c) a meniscus transports and aligns the chip; (d) droplets evaporate.
